# Chemerin and Arterial Stiffness in the Brisighella Heart Study: A Residual-Based Discordance Analysis

**DOI:** 10.3390/ijms27188264

**Published:** 2026-09-16

**Authors:** Federica Fogacci, Sergio D’Addato, Patrycja Anna Glogowski, Mirko Ragazzini, Alessandro Ciocia, Claudio Borghi, Arrigo Francesco Giuseppe Cicero, the Brisighella Heart Study Group

**Affiliations:** 1 Hypertension and Cardiovascular Risk Factors Research Centre, Medical and Surgical Sciences Department, Alma Mater Studiorum University of Bologna, 40126 Bologna, Italy; federica.fogacci@studio.unibo.it (F.F.); sergio.daddato@unibo.it (S.D.); claudio.borghi@unibo.it (C.B.); 2 Department of Medical Pharmacology, Medical Faculty, Ataturk University, 25240 Erzurum, Turkey; 3 Cardiovascular Medicine Unit, IRCCS AOU BO, 40126 Bologna, Italy; patrycja.glogowski2@unibo.it (P.A.G.);; 4 Department of Veterinary Medical Sciences, University of Bologna, 40064 Ozzano dell’Emilia, Italy

**Keywords:** chemerin, arterial stiffness, carotid–femoral pulse wave velocity, adipokines, vascular ageing, discordance analysis, Brisighella Heart Study

## Abstract

Chemerin is a multifunctional adipokine involved in metabolic regulation, inflammation, and vascular homeostasis, but its relationship with arterial stiffness remains uncertain. We investigated the association between circulating chemerin and carotid–femoral pulse wave velocity (cfPWV) and explored individual chemerin–PWV discordance in participants from the Brisighella Heart Study (*n* = 1304). Multivariable linear regression was adjusted for age, sex, body mass index, mean arterial pressure, resting heart rate, current smoking, and estimated glomerular filtration rate. Chemerin was correlated with cfPWV in unadjusted analysis (Spearman’s ρ = 0.073; *p* = 0.009). After adjustment, each one-standard-deviation increase in chemerin was associated with a nonsignificant 0.093 m/s higher cfPWV (95% confidence interval, −0.026 to 0.212; *p* = 0.126). Residual-based classification identified 132 participants with higher-than-expected chemerin and lower-than-expected cfPWV and 151 with higher-than-expected values of both measures. No investigated factor remained associated with membership in the former group after false-discovery-rate correction. Cross-fitting showed 95.6% agreement in phenotype classification. Circulating chemerin was not independently associated with central arterial stiffness, while residual analysis revealed stable but clinically unvalidated patterns of chemerin–cfPWV discordance.

## 1. Introduction

Arterial stiffening is a major feature of vascular ageing and reflects progressive functional and structural alterations of the arterial wall, including changes in extracellular matrix composition, endothelial function, and vascular smooth muscle behavior [1]. Pulse wave velocity (PWV) provides a non-invasive functional measure of this process and is widely used to quantify arterial stiffness and identify individuals with an adverse vascular phenotype [2]. Although age and blood pressure are among the principal determinants of PWV, arterial stiffening is a multifactorial process in which metabolic dysfunction, chronic inflammation, and vascular remodelling may also have an important role [3].

Adipose tissue is no longer regarded solely as an energy-storage compartment, but as a metabolically active endocrine organ that releases mediators capable of influencing immune responses, glucose and lipid metabolism, angiogenesis, and cardiovascular homeostasis [4]. Perivascular adipose tissue (PVAT), which surrounds most systemic blood vessels, is particularly relevant because its anatomical proximity to the vascular wall allows locally released factors to act directly on the endothelium and vascular smooth muscle [5]. Under physiological conditions, PVAT contributes to the regulation of vascular tone through predominantly anticontractile and vasoprotective effects [5]. In the presence of adipose-tissue dysfunction, however, its secretory profile may shift towards a pro-inflammatory and pro-contractile state, thereby promoting endothelial dysfunction and vascular remodelling [6].

Chemerin, also known as retinoic acid receptor responder 2, is a multifunctional adipokine and chemoattractant produced predominantly by adipose tissue and the liver [7]. It is initially secreted as the inactive precursor prochemerin and subsequently undergoes C-terminal proteolytic processing, generating isoforms with distinct biological activities, which may include pro-inflammatory, anti-inflammatory, contractile, or regulatory effects depending on proteolytic processing and biological context [6,7]. Chemerin acts mainly through chemokine-like receptor 1, while G protein-coupled receptor 1 and C-C chemokine receptor-like 2 contribute to the regulation of its availability and biological effects [4]. Through these pathways, chemerin participates in immune-cell recruitment, adipocyte differentiation, and the regulation of glucose and lipid metabolism [4].

A growing body of experimental evidence also supports a direct involvement of chemerin in vascular biology. Chemerin signalling may reduce endothelial nitric oxide availability, increase oxidative stress, and favour endothelial dysfunction [6]. In vascular smooth muscle cells, activation of the chemerin–CMKLR1 axis may promote calcium-dependent contraction, cellular proliferation, migration, and extracellular matrix remodelling [6]. Chemerin expression is not confined to the circulating pool, as chemerin has been detected within the vascular endothelium, smooth muscle layer, adventitia, and PVAT in experimental models [8]. The observation that pharmacological inhibition of Chemerin1 reduces vascular tone further suggests that locally produced chemerin may exert paracrine or autocrine effects within the vessel wall, independently of liver-derived circulating chemerin [8]. Chemerin-related vascular effects may also differ by sex; experimental data indicate sex-specific effects on vascular stiffness and remodeling, potentially reflecting differences in perivascular adipose-tissue distribution, local chemerin biology, and hormonal milieu [9].

Despite this biological plausibility, clinical evidence linking circulating chemerin to arterial stiffness remains inconsistent [2,10]. In older adults with type 2 diabetes, circulating chemerin concentrations were positively correlated with brachial–ankle PWV and remained independently associated with vascular stiffness in multivariable analyses [2]. A positive relationship between chemerin and aortic PWV was also reported in a small cohort of patients with morbid obesity [3]. By contrast, a study including participants with diabetes, prediabetes, and normal glucose tolerance found no independent association between serum chemerin and PWV or other markers of subclinical vascular disease [10]. These divergent findings may reflect differences in metabolic burden, study population, sample size, PWV methodology, and adjustment for demographic and hemodynamic determinants [2,10].

Previous clinical studies have primarily examined relatively small and selected populations with diabetes, prediabetes, or severe obesity [3,10]. Consequently, the relationship between circulating chemerin and central arterial stiffness in a larger community-based population remains incompletely characterized [2,3]. A community-based cohort spanning a broad spectrum of metabolic risk provides an opportunity to assess whether associations observed in selected high-risk populations extend to a broader population and whether nonlinear or threshold effects, or interindividual heterogeneity, may contribute to an apparently weak average association. Because chemerin signaling may depend on local vascular production, proteolytic processing, receptor signaling, and individual vascular susceptibility, similar circulating concentrations may not necessarily translate into uniform vascular effects [4,8]. Moreover, analyses focused exclusively on the average association between a circulating biomarker and PWV may not capture individual patterns in which the two measurements are discordant after accounting for their major clinical determinants. Conventional regression models estimate the average relationship between an exposure and an outcome at the population level, but individuals may deviate substantially from the values expected on the basis of established demographic, metabolic, or hemodynamic determinants. A residual-based approach can complement conventional analyses by quantifying these individual deviations as the difference between observed and model-predicted values. Examining residuals for both chemerin and PWV therefore provides a means of identifying subjects in whom the two phenotypes are concordant or discordant relative to their expected values, potentially revealing heterogeneity that is obscured by a single population-level regression coefficient.

Because chemerin acts within a broader inflammatory and adipokine environment, including signals arising from perivascular adipose tissue, its vascular effects may be context-dependent [4,5]. Accordingly, tumor necrosis factor-α (TNF-α), retinol-binding protein 4 (RBP4), visfatin, vaspin, and omentin-1 were evaluated as secondary exploratory markers to characterize the inflammatory and adipokine context of the chemerin–PWV residual patterns. TNF-α was of particular interest because experimental evidence indicates that it can increase chemerin expression and secretion and promote the protease-dependent generation of biologically active chemerin forms [11,12]. Against this background, the present study aimed to investigate the association between circulating chemerin and carotid–femoral PWV in a population-based sample from the Brisighella Heart Study (BHS). In addition to evaluating chemerin as a continuous variable and according to observed tertiles, we applied a residual-based approach to identify individual chemerin–PWV discordance phenotypes. We specifically examined participants with higher-than-expected chemerin but lower-than-expected PWV and compared them with participants presenting both higher-than-expected chemerin and higher-than-expected PWV. Finally, we explored the clinical, metabolic, lifestyle, and inflammatory characteristics associated with these contrasting phenotypes.

## 2. Results

### 2.1. Study Population

**Figure 1:**
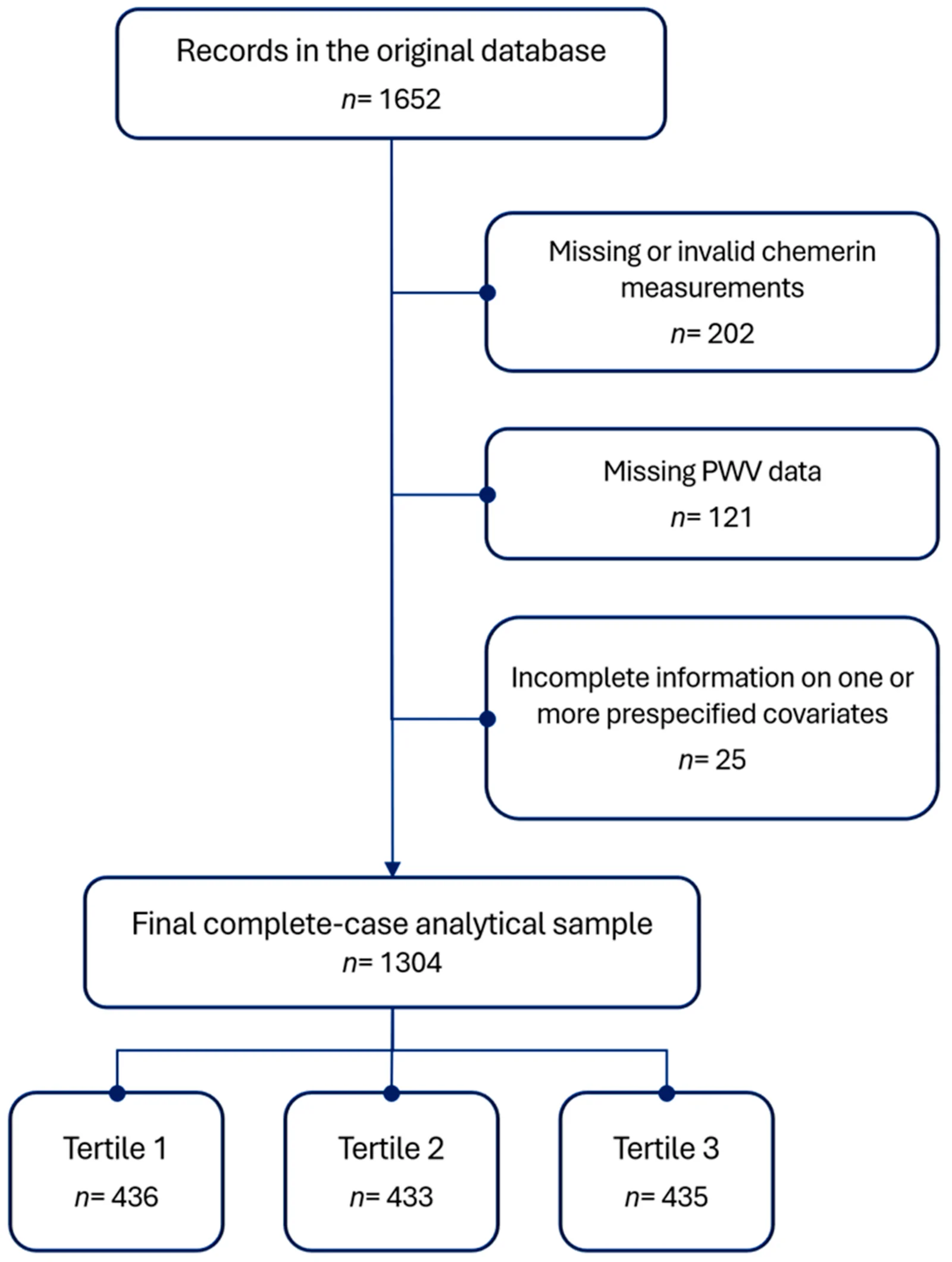
Flow-chart of participant selection. Selection of the complete-case analytical sample from the Brisighella Heart Study database.

A total of 1652 records were initially available in the BHS database. Of these, 202 were excluded because of missing or invalid chemerin measurements. Among the remaining 1450 participants, 121 had missing PWV data. A further 25 participants were excluded because of incomplete information on one or more prespecified covariates. The final complete-case analytical sample included 1304 participants, as shown in Figure 1.

Included and excluded participants were broadly comparable for most available demographic, anthropometric, metabolic, renal, and clinical characteristics, although excluded participants with available hemodynamic measurements showed somewhat higher mean arterial pressure (Supplementary Table S1).

### 2.2. Characteristics According to Observed Chemerin Tertiles

The cut-off points for the observed chemerin tertiles were 272.65 ng/mL and 371.77 ng/mL. The lowest, middle, and highest tertiles included 436, 433, and 435 participants, respectively.

The median age of the analytical population was 58.0 years [interquartile range, 46.8–71.0]; 676 participants were women (51.8%); and the median body mass index was 26.3 kg/m^2^ [23.6–29.4]. Median waist circumference was 93.0 cm [83.0–101.0], and median PWV was 8.7 m/s [7.6–10.2].

Demographic, anthropometric, hemodynamic, renal, and metabolic characteristics were generally comparable across chemerin tertiles. No statistically significant differences were observed in age, sex distribution, body mass index, waist circumference, systolic or diastolic blood pressure, mean arterial pressure, resting heart rate, estimated glomerular filtration rate, fasting glucose, high-density lipoprotein (HDL) cholesterol, low-density lipoprotein (LDL) cholesterol, current smoking, known hypertension, known diabetes, or cardiovascular and glucose-lowering treatments (Table 1).

PWV showed a modest increase across chemerin tertiles. Median PWV was 8.5 m/s [7.5–9.9] in the lowest tertile, 8.8 m/s [7.7–10.4] in the middle tertile, and 8.9 m/s [7.7–10.3] in the highest tertile (*p* = 0.019). Mean PWV values were 8.90, 9.18, and 9.24 m/s, respectively.

### 2.3. Inflammatory and Adipokine Profiles Across Chemerin Tertiles

The distribution of selected inflammatory and adipokine biomarkers differed across observed chemerin tertiles (Supplementary Table S2).

TNF-α concentrations were higher in the highest chemerin tertile. Median values were 5.99 pg/mL [3.25–9.11], 5.98 pg/mL [3.80–8.70], and 7.13 pg/mL [4.30–9.79] across increasing chemerin tertiles, respectively (*p* < 0.001; false-discovery-rate-adjusted q = 0.001). Conversely, RBP4 concentrations decreased across chemerin tertiles, with median values of 54 µg/mL [39.5–69.43], 47.25 µg/mL [35.52–65.9], and 45.6 µg/mL [35.55–65.26], respectively (*p* < 0.001; q < 0.001). A similar inverse pattern was observed for visfatin.

Median visfatin concentrations were 5.95 ng/mL [4.83–7.52], 5.92 ng/mL [4.57–7.70], and 5.42 ng/mL [4.44–6.88] across increasing chemerin tertiles, respectively (*p* < 0.001; q < 0.001).

No statistically significant differences were observed for vaspin or omentin-1 after false-discovery-rate correction.

### 2.4. Association Between Circulating Chemerin and PWV

**Figure 2:**
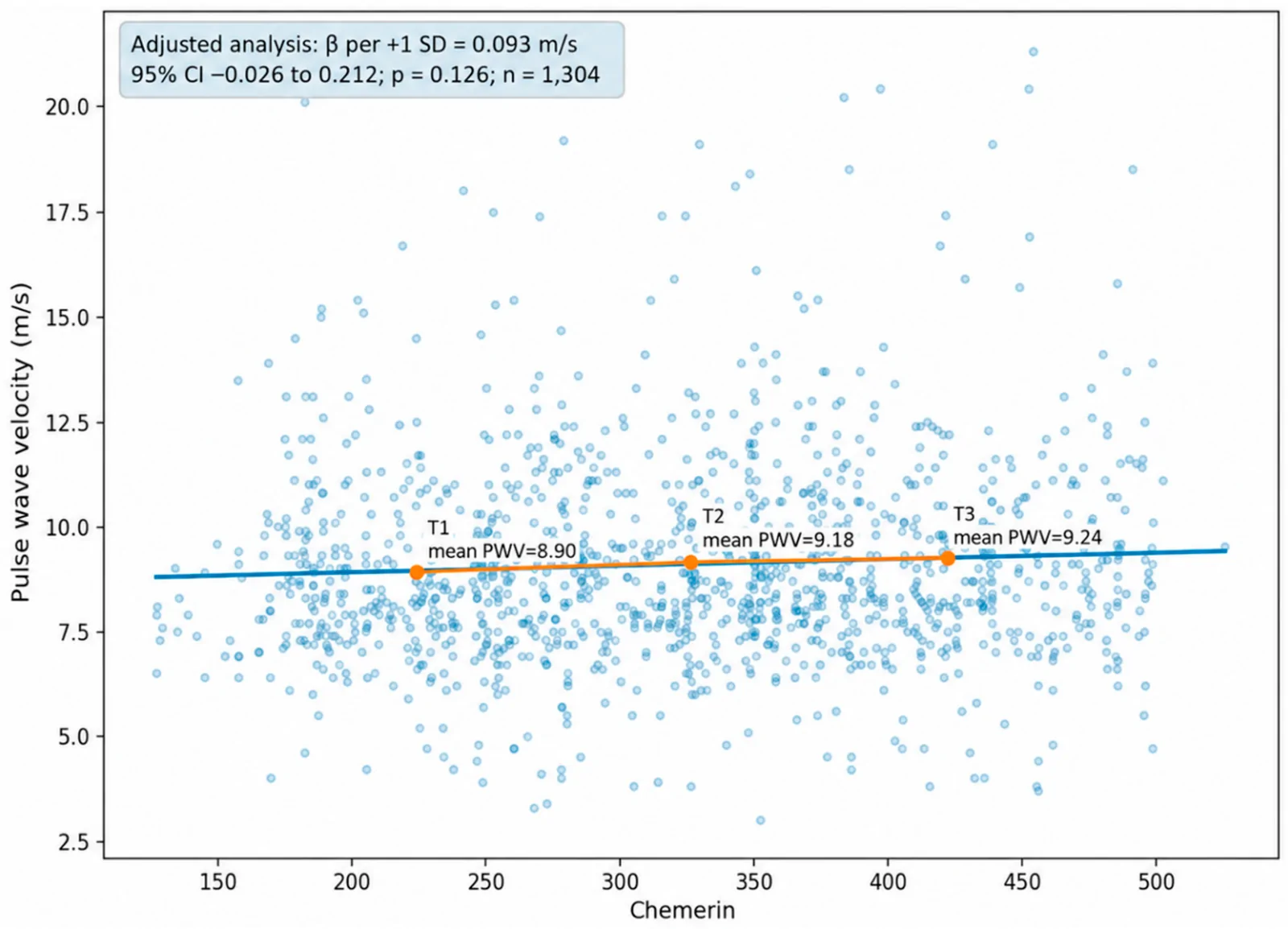
Association between circulating chemerin and pulse wave velocity. Scatterplot showing the relationship between circulating chemerin and pulse wave velocity in the complete-case analytical sample. Each point represents one individual. The blue solid line represents the unadjusted linear regression fit. Orange markers indicate the mean PWV values plotted at the median chemerin value within each tertile of observed chemerin, and the orange line connects these points to illustrate the distributional pattern. In the multivariable model adjusted for age, sex, body mass index, mean arterial pressure, resting heart rate, current smoking status, and estimated glomerular filtration rate, each one-standard-deviation increase in chemerin was associated with a 0.093 m/s higher PWV (95% confidence interval, −0.026 to 0.212; *p* = 0.126). Abbreviations: CI, confidence interval; PWV, pulse wave velocity; SD, standard deviation.

In the unadjusted analysis, circulating chemerin was weakly but positively correlated with PWV (Spearman’s ρ = 0.073; *p* = 0.009; Figure 2).

The mean chemerin concentration in the analytical sample was 323.6 ± 91.6 ng/mL. After adjustment for age, sex, body mass index, mean arterial pressure, resting heart rate, current smoking status, and estimated glomerular filtration rate, each one-standard-deviation increase in chemerin was associated with a 0.093 m/s higher PWV. However, the confidence interval (CI) included the null value, and the association was not statistically significant (β = 0.093 m/s; 95% CI, −0.026 to 0.212; *p* = 0.126). The adjusted model explained 23.1% of the variability in PWV. Modeling chemerin using a natural cubic spline provided no evidence of departure from linearity (*p* for nonlinearity = 0.342), and the overall spline association was not statistically significant (*p* = 0.141).

When chemerin was examined according to tertiles, the adjusted difference in PWV was 0.205 m/s for the middle versus the lowest tertile (*p* = 0.137) and 0.228 m/s for the highest versus the lowest tertile (*p* = 0.120). There was no statistically significant linear trend across the three tertiles (β per tertile increase = 0.114 m/s; *p* for trend = 0.120).

Thus, the modest unadjusted increase in PWV across chemerin tertiles was attenuated after adjustment for the prespecified demographic, hemodynamic, metabolic, and renal covariates.

### 2.5. Chemerin–PWV Discordance Phenotypes

**Figure 3:**
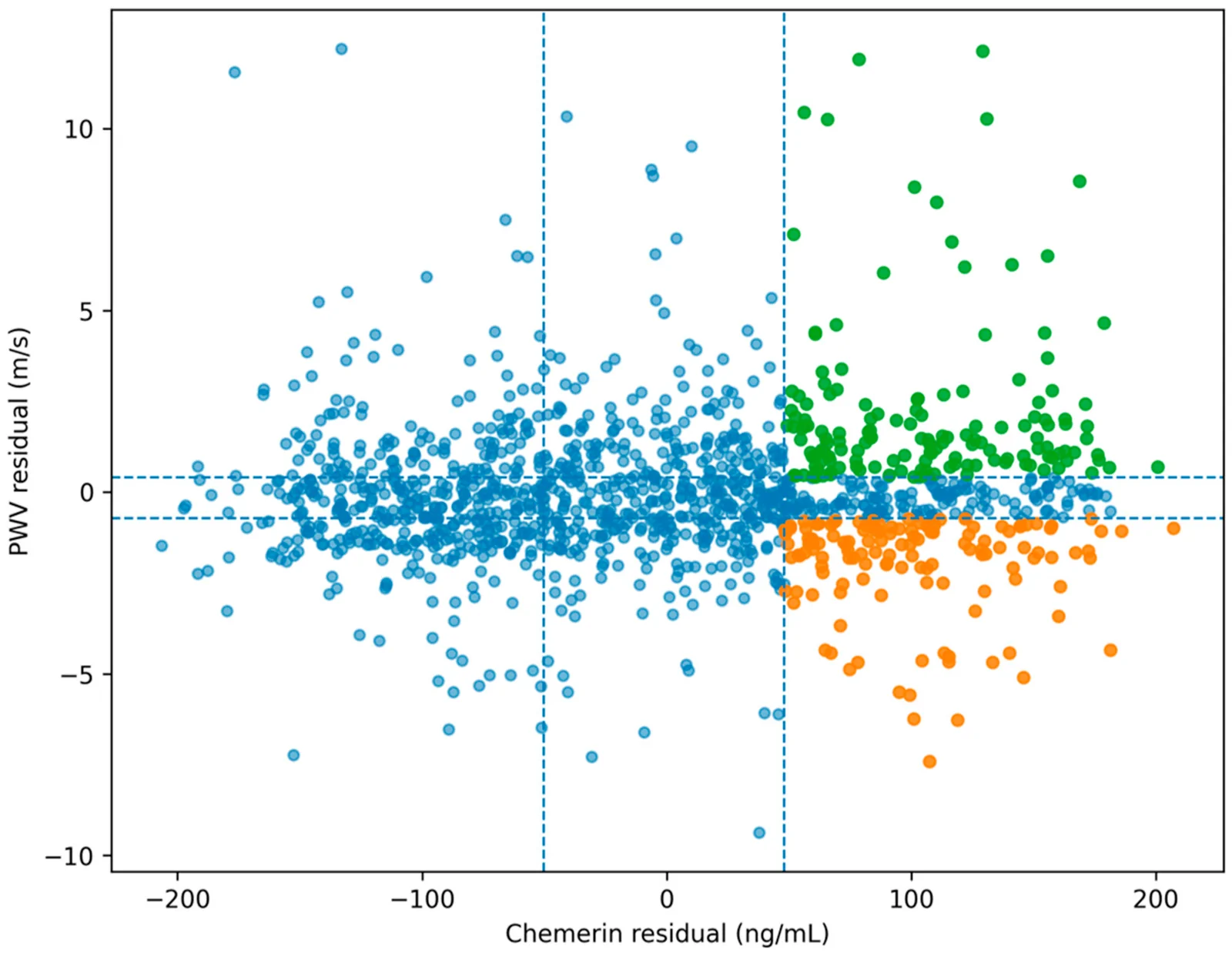
Individual-level chemerin–PWV discordance map. Individual distribution of chemerin and pulse wave velocity residuals in the analytical sample. Each point represents one subject. Chemerin residuals were calculated as observed minus predicted chemerin values from a model including age, sex, body mass index, and estimated glomerular filtration rate. PWV residuals were calculated as observed minus predicted PWV values from a model including age, sex, body mass index, mean arterial pressure, resting heart rate, current smoking status, and eGFR. Dashed lines indicate the tertile cut-off points of each residual distribution and define nine chemerin–PWV phenotype cells. Orange points indicate participants with higher-than-expected chemerin and lower-than-expected PWV; green points indicate participants with higher-than-expected values of both measures; and blue points represent the remaining residual patterns. Abbreviation: PWV, pulse wave velocity.

**Figure 4:**
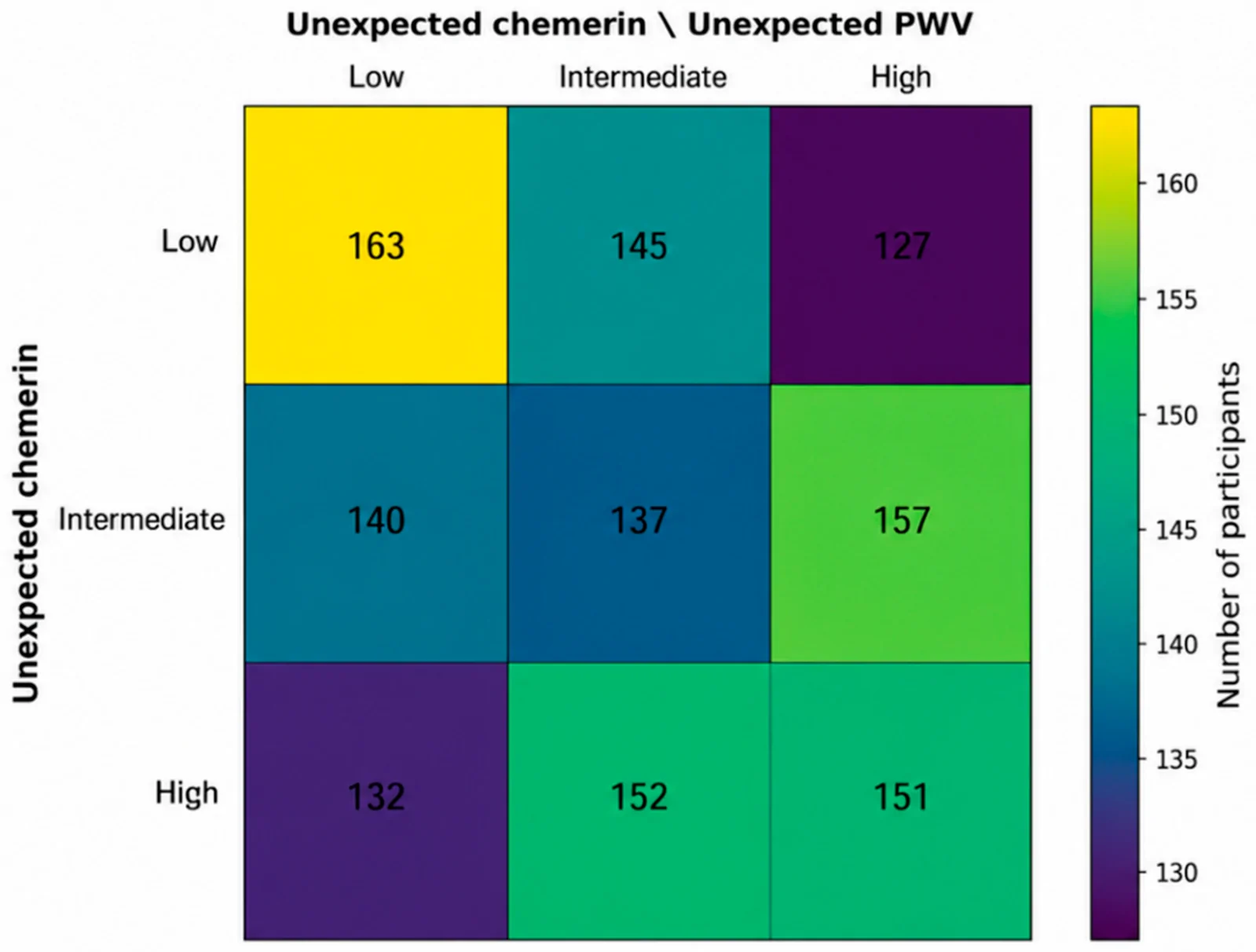
Three-by-three classification of chemerin–PWV residual patterns. Three-by-three matrix obtained by independently dividing chemerin and PWV residuals into tertiles. Rows represent tertiles of chemerin residuals, and columns represent tertiles of PWV residuals. Cell values indicate the number of participants assigned to each residual pattern. The bottom-left cell represents participants with higher-than-expected chemerin and lower-than-expected PWV (*n* = 132), whereas the bottom-right cell represents participants with higher-than-expected values of both chemerin and PWV (*n* = 151). The middle tertiles were retained as intermediate categories to reduce the classification of participants with residual values close to the distributional cut-off points as extreme phenotypes. Abbreviation: PWV, pulse wave velocity.

Chemerin and PWV residuals were independently divided into tertiles, resulting in nine discordance phenotypes (Figure 3 and Figure 4).

The complete distribution of participants across the 3 × 3 residual matrix is shown in Figure 4. Among those with chemerin residuals in the highest tertile, 132 had PWV residuals in the lowest tertile, 152 in the middle tertile, and 151 in the highest tertile. Accordingly, the two extreme groups selected for further comparison comprised 132 participants with higher-than-expected chemerin and lower-than-expected PWV and 151 participants with higher-than-expected values of both measures. The corresponding individual-level distribution across the chemerin–PWV residual plane is shown in Figure 3.

### 2.6. Characteristics of the Contrasting High-Chemerin Residual Groups

Known diabetes was less prevalent among participants with higher-than-expected chemerin and lower-than-expected PWV than among those with higher-than-expected values of both measures (5.4% vs. 12.2%; *p* = 0.047).

Participants in the former group also tended to be older (median age, 64 vs. 59 years; *p* = 0.052), to have a greater waist circumference (94.5 vs. 91.0 cm; *p* = 0.081), to have higher LDL cholesterol concentrations (141.4 vs. 135.8 mg/dL; *p* = 0.084), and to be less frequently current smokers (11.4% vs. 18.5%; *p* = 0.093).

However, none of these unadjusted differences remained statistically significant after false-discovery-rate correction, with adjusted q-values of at least 0.465 (Table 2).

### 2.7. Factors Associated with the Higher-than-Expected Chemerin/Lower-than-Expected PWV Phenotypes

No meaningful between-group differences were observed in body mass index, mean arterial pressure, resting heart rate, estimated glomerular filtration rate (eGFR), fasting glucose, triglycerides, HDL cholesterol, or antihypertensive treatment. Similarly, circulating TNF-α, RBP4, visfatin, vaspin, and omentin-1 concentrations did not significantly distinguish between the two residual-based groups after multiple-testing correction (Table 2).

**Figure 5:**
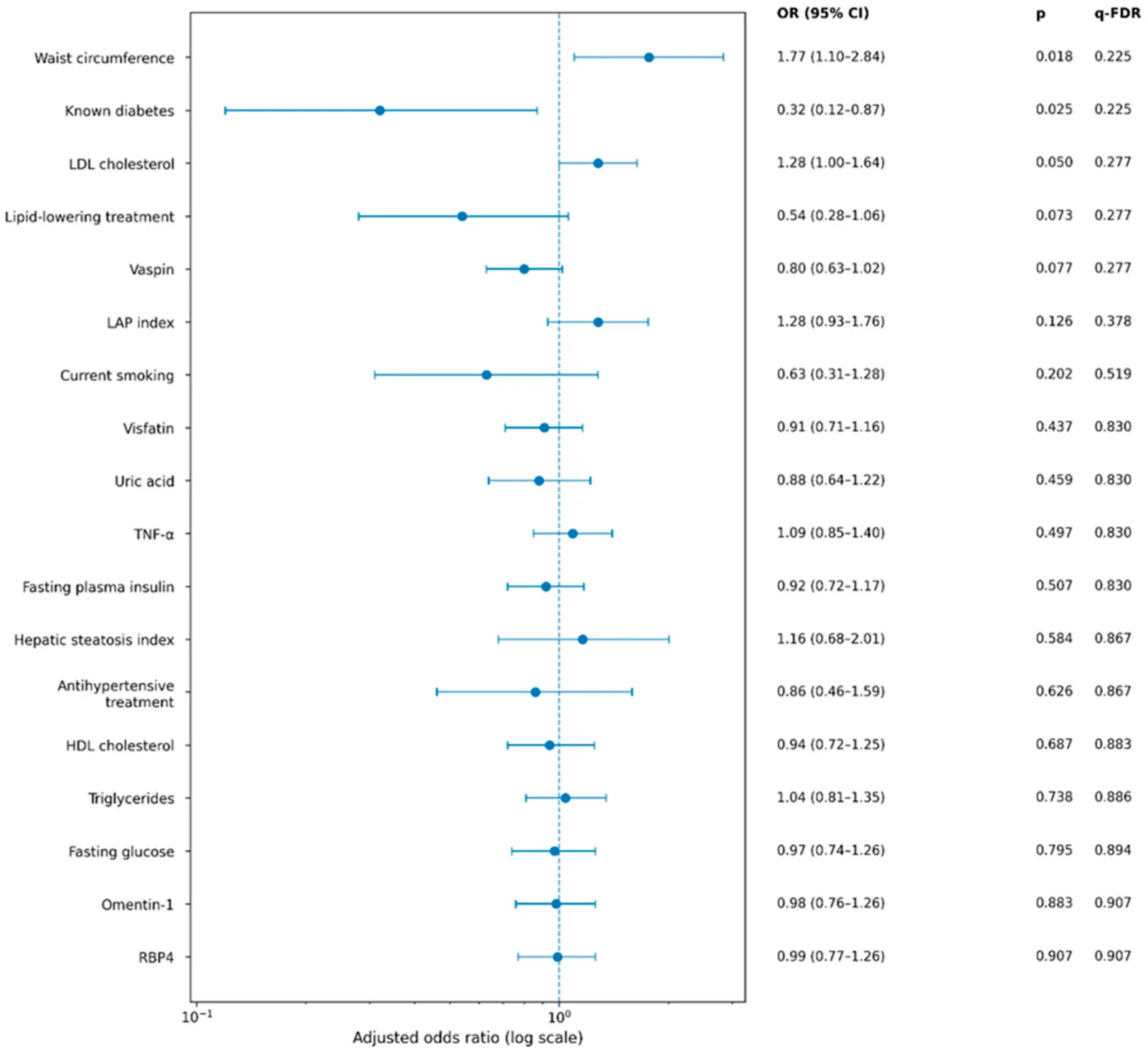
Factors associated with the higher-than-expected chemerin/lower-than-expected PWV phenotype. Forest plot showing odds ratios and 95% confidence intervals for factors potentially associated with membership in the higher-than-expected chemerin/lower-than-expected PWV rather than the higher-than-expected chemerin/higher-than-expected PWV group. Each candidate factor was evaluated in a separate logistic regression model adjusted for age, sex, body mass index, mean arterial pressure, resting heart rate, current smoking status, and estimated glomerular filtration rate. Age and mean arterial pressure were modeled using centered natural cubic splines. Continuous candidate variables were standardized, and odds ratios are expressed per one-standard-deviation increase. The vertical reference line indicates an odds ratio of 1. None of the evaluated factors remained statistically significant after correction for multiple comparisons using the Benjamini–Hochberg false-discovery-rate procedure. Abbreviations: CI, confidence interval; FDR, false discovery rate; OR, odds ratio; PWV, pulse wave velocity.

In covariate-adjusted exploratory logistic regression analyses, a one-standard-deviation greater waist circumference was associated with higher odds of belonging to the higher-than-expected chemerin/lower-than-expected PWV group rather than the group with higher-than-expected values of both measures (odds ratio, 1.77; 95% CI, 1.10–2.84; *p* = 0.018) (Figure 5).

Known diabetes was associated with lower odds of belonging to the higher-than-expected chemerin/lower-than-expected PWV group (odds ratio, 0.32; 95% CI, 0.12–0.87; *p* = 0.025).

Higher LDL cholesterol showed a borderline positive association with membership in this group (odds ratio per one-standard-deviation increase: 1.28; 95% CI, 1.00–1.64; *p* = 0.050). Lipid-lowering treatment and higher vaspin concentrations showed nonsignificant inverse associations, with odds ratios of 0.54 (95% CI, 0.28–1.06; *p* = 0.073) and 0.80 per one-standard-deviation increase (95% CI, 0.63–1.02; *p* = 0.077), respectively.

None of these associations remained statistically significant after false-discovery-rate correction (Table 3). The adjusted q-values were 0.225 for waist circumference and known diabetes and 0.277 for LDL cholesterol, lipid-lowering treatment, and vaspin. No significant adjusted associations were observed for tumor necrosis factor-α or the other evaluated adipokines.

### 2.8. Robustness of Residual-Based Findings

The residual-based phenotype classification was highly stable in the 10-fold cross-fitting analysis. Overall, 98.3% of participants remained in the same chemerin residual tertile, 97.2% remained in the same PWV residual tertile, and 95.6% remained in the same cell of the 3 × 3 discordance matrix (Supplementary Table S3). Phenotype assignment was also robust to harmonization of the covariate sets used to estimate expected chemerin and PWV. When the expected-chemerin model was refitted using the same covariate set as the expected-PWV model, 94.8% of participants remained in the same 3 × 3 phenotype cell. The higher-than-expected chemerin/lower-than-expected PWV and higher-than-expected chemerin/higher-than-expected PWV groups included 133 and 151 participants, respectively, with 97.7% and 96.0% of participants originally assigned to these extreme groups retaining their classification (Supplementary Table S3).

Additional adjustment for known diabetes, known hypertension, lipid-lowering treatment, or TNF-α, as well as replacement of body mass index with waist circumference, did not materially alter the association between circulating chemerin and PWV. Across these sensitivity analyses, the adjusted coefficient ranged from 0.082 to 0.102 m/s per one-standard-deviation increase in chemerin, and none of the associations was statistically significant. In the extended model incorporating all additional factors, the association remained similar to the primary estimate (β = 0.091 m/s; 95% confidence interval, −0.031 to 0.213; *p* = 0.142; *n* = 1214) (Supplementary Table S4). The residual-based findings were also insensitive to the choice of classification thresholds. Median-based classification identified 313 participants with higher-than-expected chemerin and lower-than-expected PWV and 339 with higher-than-expected values of both measures. Using the more restrictive quartile-based classification, the corresponding extreme groups included 74 and 81 participants, respectively. Repeating the phenotype-characterization comparisons under either alternative classification did not identify any between-group difference that remained statistically significant after false-discovery-rate correction (minimum q-FDR = 0.672 for the median-based classification and 0.670 for the quartile-based classification) (Supplementary Table S3).

## 3. Discussion

In this population-based analysis of the BHS, circulating chemerin showed a weak positive relationship with pulse wave velocity in unadjusted analyses. PWV increased modestly across chemerin tertiles, and chemerin was positively correlated with PWV when the two variables were considered without covariate adjustment. However, the association was substantially attenuated after accounting for age, sex, body mass index, mean arterial pressure, resting heart rate, smoking status, and renal function. In the fully adjusted model, a one-standard-deviation increase in chemerin was associated with only a small, statistically nonsignificant increase in PWV. The consistency of this finding across additional models accounting for diabetes, hypertension, central adiposity, lipid-lowering treatment, and systemic inflammation further supports the robustness of the primary result. Thus, there was no statistically significant independent association between circulating chemerin and central arterial stiffness after adjustment for the prespecified clinical and hemodynamic determinants.

Our findings contribute to the clinical literature, which has produced heterogeneous results. In older adults with type 2 diabetes, Li et al. reported a strong positive correlation between chemerin and brachial–ankle PWV and identified chemerin as an independent correlate of vascular stiffness [2]. A positive association between chemerin and aortic PWV was also observed in a small study of patients with morbid obesity, in whom metabolic abnormalities and adipose-tissue dysfunction were particularly pronounced [3]. Conversely, Aydın et al. found no association between serum chemerin and PWV, carotid intima–media thickness, epicardial fat thickness, or carotid plaque after adjustment in participants with diabetes, prediabetes, or normal glucose tolerance [10]. Our results are therefore more consistent with the latter study, although they extend the evidence to a considerably larger, community-based sample.

Several features may explain the discrepancies among available studies. Previous positive investigations were conducted in older adults with diabetes, patients with morbid obesity, or individuals with essential hypertension, whereas the present cohort included individuals across a broader spectrum of age, adiposity, metabolic health, and cardiovascular risk [2,3,13]. Chemerin-related vascular effects may become more apparent in settings characterized by marked insulin resistance, dysfunctional adipose tissue, chronic inflammation, or severe metabolic disease. In addition, studies have used different measures of arterial stiffness, including brachial–ankle PWV, aortic PWV estimated through oscillometric methods, and directly measured carotid–femoral PWV. These approaches reflect overlapping but not identical vascular territories and may differ in their sensitivity to peripheral vascular disease, blood pressure, and metabolic abnormalities.

The attenuation observed after multivariable adjustment is also informative. Age and blood pressure are major determinants of PWV, and both variables are strongly related to vascular structure and function [2]. The modest unadjusted relationship between chemerin and PWV may therefore partly reflect their shared associations with ageing, hemodynamic load, adiposity, renal function, and other cardiovascular risk factors. This interpretation is supported by previous clinical studies in which chemerin was associated with several components of the metabolic syndrome but did not independently predict the vascular outcome after comprehensive adjustment [10]. Rather than acting as a robust standalone marker of arterial stiffness, total circulating chemerin may therefore integrate several metabolic and inflammatory processes that also influence PWV.

Nevertheless, the absence of a statistically significant independent association does not exclude the role of chemerin in vascular biology. Experimental evidence indicates that chemerin can impair nitric oxide signaling, increase oxidative stress, enhance vasoconstrictor responses, and promote vascular smooth muscle cell proliferation and migration [4,14,15]. Importantly, circulating total chemerin may not accurately reflect biologically active chemerin within the vascular wall. Chemerin is expressed in vascular smooth muscle, endothelium, adventitia, and perivascular adipose tissue, where it may exert local autocrine or paracrine effects [8,16]. In addition, proteolytic processing generates chemerin isoforms with markedly different biological activities, whereas conventional assays quantify total circulating chemerin [17]. These features may weaken the relationship between measured plasma concentrations and vascular outcomes and may partly explain why experimental vascular effects do not translate into a strong association with central arterial stiffness in the present population.

The inflammatory and adipokine patterns observed across chemerin tertiles further illustrate this biological complexity. Participants in the highest chemerin tertile had higher TNF-α concentrations, consistent with experimental evidence linking TNF-α to increased chemerin expression, secretion, and protease-dependent activation [11,12,18]. However, RBP4 and visfatin concentrations decreased across increasing chemerin tertiles, whereas vaspin and omentin-1 did not differ significantly. Thus, higher circulating chemerin did not identify a uniformly adverse inflammatory or adipokine profile. Context-dependent relationships have also been reported in advanced carotid atherosclerosis, where circulating chemerin was not independently associated with cholesterol efflux capacity and its associations varied according to the accompanying inflammatory and adipokine milieu [19]. Together, these observations support the interpretation of circulating chemerin as a context-dependent biomarker rather than a uniform marker of adverse vascular biology.

A distinctive aspect of the present study was the residual-based analysis of chemerin–PWV discordance. By estimating values expected from the principal clinical determinants of each measure, this approach identified 132 participants with higher-than-expected chemerin but lower-than-expected PWV and 151 with higher-than-expected values of both measures. Cross-fitting supported the internal statistical stability of this classification, but does not establish its biological validity, prognostic significance, or clinical utility. At present, these residual patterns should therefore not be used for cardiovascular risk stratification, treatment decisions, or patient management. Their potential usefulness is primarily hypothesis-generating, by identifying individuals whose circulating chemerin and vascular stiffness differ from what would be expected on the basis of conventional clinical determinants. Clinical application would require external replication and prospective evidence that these patterns predict progression of arterial stiffness or cardiovascular outcomes beyond established risk factors. In particular, the higher-than-expected chemerin/lower-than-expected PWV pattern should not be interpreted as evidence that higher chemerin is protective. Rather, it highlights individual heterogeneity that could reflect differences in chemerin isoforms, receptor signaling, local vascular production, exposure duration, or arterial susceptibility.

Sex-related heterogeneity may also contribute to this. Experimental chemerin knockout data indicate sex-specific effects on PWV, vascular remodeling, and obesity-associated hypertension [9]. Although sex was included as a covariate in our models, the present analysis was not designed to assess sex-specific chemerin–PWV relationships, and this possibility warrants investigation in appropriately powered studies.

In exploratory comparisons, known diabetes was less frequent among participants with higher-than-expected chemerin and lower-than-expected PWV, whereas waist circumference and LDL cholesterol tended to be higher. After adjustment, greater waist circumference and the absence of known diabetes showed nominal associations with membership in this group. Although the lower prevalence of diabetes is directionally consistent with the established association between diabetes and vascular stiffening [2], the present finding derives from an exploratory residual-based analysis and should not be interpreted as confirmatory evidence. The association with greater waist circumference is less intuitive and may reflect residual confounding or chance. Importantly, none of these associations remained statistically significant after false-discovery-rate correction; these findings should therefore be regarded as hypothesis-generating rather than established associations. Future studies integrating isoform-specific chemerin measurements, vascular imaging, and longitudinal vascular outcomes may clarify whether these residual patterns have biological or clinical relevance.

### Strengths and Limitations

The present study has several strengths. It was conducted in a relatively large, community-based cohort rather than in a small, highly selected clinical group. Arterial stiffness was evaluated using carotid–femoral PWV, and the primary analysis incorporated major demographic, anthropometric, hemodynamic, behavioral, and renal determinants. Potential nonlinear associations of age and mean arterial pressure were addressed using natural cubic splines, while heteroskedasticity-consistent standard errors were used for statistical inference. The continuous, tertile-based, and residual-based approaches provided complementary perspectives on the chemerin–PWV relationship. Finally, cross-fitting demonstrated high stability of the residual phenotype classification, and false-discovery-rate correction was applied to the exploratory analyses.

Several limitations should also be acknowledged. First, the cross-sectional design precludes conclusions regarding temporality or causality. Chemerin and PWV were measured during the same survey, and it cannot be established whether chemerin contributes to vascular stiffening, reflects concurrent metabolic or inflammatory processes, or is influenced by vascular disease.

Second, chemerin was measured at a single time point. Intraindividual biological variability and changes related to acute inflammation, metabolic status, medication use, or sample handling may have introduced measurement error. Such nondifferential variability would be expected to attenuate the estimated association.

Third, the assay quantified total circulating chemerin and did not distinguish between inactive prochemerin and bioactive or regulatory isoforms. Because chemerin isoforms differ substantially in receptor activity and may be generated locally by tissue-specific proteases, total plasma chemerin may be an incomplete proxy for biologically relevant chemerin signaling [17].

Fourth, circulating chemerin does not necessarily reflect chemerin production or activity within the vascular wall and perivascular adipose tissue. Experimental evidence indicates that the vasculature may produce chemerin independently of the liver and that locally produced chemerin contributes to vascular tone [8]. No direct measurements of vascular or perivascular chemerin, receptor expression, endothelial function, or extracellular matrix composition were available in the present study.

Fifth, although the primary models accounted for several major determinants of PWV, residual confounding cannot be excluded. Hormonal status, detailed body-fat distribution, dietary factors, inflammatory conditions, duration and control of diabetes or hypertension, and other pharmacological exposures may influence both chemerin and vascular stiffness. Adjustment for clinical covariates also cannot fully account for differences in lifetime exposure to blood pressure or metabolic disease.

Sixth, the discordance phenotypes were data-derived and depended on the selected prediction models and tertile cut-off points. Although their internal statistical stability was supported by cross-fitting, they have not been biologically, clinically, or externally validated and should therefore be interpreted as descriptive statistical patterns rather than cardiovascular-risk categories. Longitudinal follow-up is required to determine their prognostic relevance.

Seventh, the phenotype-characterization analyses involved multiple candidate factors and relatively small numbers of participants in the two extreme groups. None of the observed associations remained significant after false-discovery-rate correction, and the estimates may be unstable. These analyses should therefore be regarded as exploratory.

Eighth, complete-case analysis may have introduced selection bias. Although included and excluded participants were broadly comparable for most available characteristics, excluded participants with available hemodynamic measurements had somewhat higher mean arterial pressure, and missing data limited the completeness of this comparison. Finally, the BHS population was recruited from a rural municipality in Northern Italy, and the findings may not be directly generalizable to populations with different ethnic, socioeconomic, metabolic, or cardiovascular-risk profiles.

## 4. Methods

### 4.1. Study Framework and Participant Selection

The BHS was established in 1972 as a community-based investigation of cardiovascular health in Brisighella, a rural municipality in Northern Italy. At study inception, 2939 residents without clinically evident cardiovascular disease were randomly selected to provide a sample representative of the local population. Follow-up surveys were subsequently conducted at approximately four-year intervals, enabling the repeated collection of clinical measurements, biochemical data, and information on cardiovascular risk factors, treatments, and health outcomes [20].

The present investigation was a secondary, retrospective, cross-sectional analysis of data collected during the BHS survey conducted between 2020 and 2023. Circulating chemerin, carotid–femoral PWV (cfPWV), clinical characteristics, and laboratory variables were assessed during the same examination; therefore, the analyses were intended to evaluate contemporaneous associations rather than prospective relationships.

Participants were eligible when valid measurements of circulating chemerin and cfPWV were available, together with complete information on the prespecified covariates required for the primary multivariable model. No additional disease-based exclusion criteria were applied specifically for this secondary analysis; participant selection was based on the availability and validity of the required chemerin and cfPWV measurements and complete data for the prespecified covariates.

Data quality and internal consistency were evaluated before statistical analysis. Measurements considered incompatible with physiological or analytical plausibility were treated as missing or excluded according to the prespecified criteria described in the statistical analysis section. The participant selection process is summarized in Figure 1. To assess potential selection bias, the main available characteristics of included and excluded participants were compared using standardized mean differences, based on all available observations for each variable.

The overall BHS protocol was approved by the Institutional Ethics Committee of the IRCCS Azienda Ospedaliero-Universitaria di Bologna (BrixFollow-up_1972-2024; PI Prof. Arrigo F.G. Cicero). The study was conducted in accordance with the principles of the Declaration of Helsinki, and all participants provided written informed consent.

### 4.2. Clinical and Anthropometric Assessment

Clinical information was collected by trained study personnel through a standardized interview covering personal and family medical history, smoking habits, lifestyle, and current medication use. Smoking status was classified as never, former, or current smoking. The interview was followed by a physical examination performed according to established BHS procedures [21,22].

Standing height was measured without shoes, while body weight was recorded twice using a calibrated scale; the mean of the two measurements was used for analysis. Body mass index was calculated as weight in kilograms divided by height in meters squared. Waist circumference was measured in the abdominal region midway between the lower costal margin and the iliac crest [21].

Peripheral blood pressure was measured using an automated electronic sphygmomanometer with a cuff selected according to arm circumference. Participants rested quietly in the seated position for at least 5 min before the measurement. Three consecutive readings were then obtained at 1-min intervals, and the mean systolic and diastolic blood pressure values were retained for analysis [22,23].

Mean arterial pressure was calculated as diastolic blood pressure plus one-third of pulse pressure. Resting heart rate was recorded during the same standardized assessment [23].

### 4.3. Biochemical and Biomarker Measurements

Venous blood samples were collected from an antecubital vein between 08:00 and 09:00 after a 12-h overnight fast, with fasting status confirmed by participant report before venipuncture. Blood was drawn using a 19-gauge needle, with care taken to minimize venous stasis, and collected into tubes containing Na_2_-EDTA at a final concentration of 1 mg/mL. Plasma was separated by a single centrifugation at 3000× *g* for 15 min at 4 °C, aliquoted directly without a second centrifugation, immediately frozen, and stored at −80 °C until analysis. Laboratory measurements were performed at a central laboratory using standardized procedures.

The conventional biochemical profile included fasting plasma glucose, total cholesterol, triglycerides, high-density lipoprotein cholesterol, low-density lipoprotein cholesterol, creatinine, hepatic enzymes, and uric acid [24]. Routine biochemical measurements were performed using a cobas c 311 automated analyzer (Roche Diagnostics GmbH, Mannheim, Germany). Renal function was estimated using the Chronic Kidney Disease Epidemiology Collaboration equation [25].

The advanced biomarker panel included TNF-α, RBP4, visfatin, vaspin, omentin-1, and chemerin.

TNF-α concentrations were measured using a commercially available enzyme-linked immunosorbent assay (Titer-Zyme EIA kit; Assay Designs, Ann Arbor, MI, USA), in accordance with the manufacturer’s instructions. The minimum analytical detection limit was 8.43 pg/mL. The intra-assay coefficients of variation were 4.5% at low concentrations and 3.6% at high concentrations, while the corresponding inter-assay coefficients of variation were 6.0% and 11.8%.

RBP4 was measured using a human enzyme immunoassay (RBP-4 Human EIA kit; Phoenix Pharmaceuticals, Burlingame, CA, USA). The minimum analytical detection limit was 1.4 ng/mL. The intra-assay coefficient of variation was below 5.0%, and the inter-assay coefficient of variation was below 14.0%.

Vaspin concentrations were assessed using a two-site enzyme-linked immunosorbent assay (AdipoGen, Seoul, Republic of Korea). The minimum analytical detection limit was 12 pg/mL. The intra-assay and inter-assay coefficients of variation were 1.74% and 8.32%, respectively [26].

Visfatin was quantified using a commercially available enzyme immunoassay kit (Phoenix Pharmaceuticals, Burlingame, CA, USA). The minimum analytical detection limit was 2.3 ng/mL. The intra-assay coefficient of variation was 10%, while the inter-assay coefficient of variation was below 14% [27].

Omentin-1 concentrations were measured using a two-site commercial enzyme-linked immunosorbent assay (Enzo Life Sciences, Farmingdale, NY, USA). The minimum analytical detection limit was 0.4 ng/mL. The intra-assay and inter-assay coefficients of variation were 7.4% and 4.77%, respectively [28].

Chemerin concentrations were determined using a commercial enzyme-linked immunosorbent assay (BioVendor, Heidelberg, Germany). The minimum analytical detection limit was 0.1 ng/mL. The intra-assay and inter-assay coefficients of variation were below 6% and 8%, respectively.

Before analysis, all biomarker measurements underwent plausibility and consistency checks. Values considered analytically implausible were treated as missing. Analyses involving biomarkers other than chemerin were restricted to participants with a valid measurement of the biomarker under consideration.

### 4.4. Measurement of Carotid–Femoral Pulse Wave Velocity

CfPWV was measured non-invasively using the Vicorder^®^ system (Skidmore Medical Ltd., Bristol, UK). This cuff-based device records carotid and femoral pulse waveforms simultaneously and has been validated against established methods for the assessment of aortic arterial stiffness, with satisfactory measurement accuracy and repeatability [29,30].

Measurements were performed with participants resting in the supine position. A partially inflatable cuff was placed around the neck over the right common carotid artery, taking care to avoid compression of the trachea or simultaneous compression of both carotid arteries. A second cuff was positioned as proximally as possible around the right upper thigh to record the femoral pulse waveform. Device-specific Vicorder^®^ cuffs were used for carotid and femoral waveform acquisition. For participants with larger thigh circumferences, an appropriately sized larger thigh cuff was selected to ensure adequate fitting.

The carotid–femoral propagation distance was measured over the body surface with a flexible tape, from the suprasternal notch to the upper edge of the thigh cuff, consistent with the Vicorder^®^ measurement approach described in the validation study [29], and entered directly into the device software. No multiplicative correction factor was applied to the measured distance.

The carotid and femoral cuffs were then inflated simultaneously to approximately 65 mmHg, allowing synchronous acquisition of the two oscillometric waveforms over a sequence of stable cardiac cycles.

Pulse transit time was calculated automatically from the temporal delay between the carotid and femoral waveforms using the integrated waveform-analysis algorithm. The device subsequently calculated cfPWV as the ratio between the measured propagation distance and pulse transit time, with results expressed in meters per second.

Waveform morphology, signal stability, and the absence of movement artefacts were visually assessed before each recording was accepted. Acquisitions affected by signal noise, participant movement, or unstable waveforms were repeated. At least three technically satisfactory waveform sequences were obtained. Two cfPWV estimates were considered reproducible when they differed by no more than 0.5 m/s, and their mean was retained as the participant’s final value. If this criterion was not met, the measurement procedure was repeated [31].

For simplicity, cfPWV is referred to as PWV. In the present analysis, PWV was treated as a continuous measure of arterial stiffness rather than dichotomized according to a clinical threshold. As part of data cleaning, values between 3 and 25 m/s were considered physiologically plausible.

### 4.5. Statistical Analysis

Continuous variables were summarized as median and interquartile range, whereas categorical variables were reported as counts and percentages. The distributions of continuous variables were assessed before analysis. No missing-data imputation was performed, and the primary analyses were based on complete cases.

For descriptive purposes, participants were categorized according to tertiles of the observed chemerin distribution. Differences in demographic, anthropometric, hemodynamic, renal, metabolic, and treatment-related characteristics across chemerin tertiles were assessed using the Kruskal–Wallis test for continuous variables and the chi-square test for categorical variables. Fisher’s exact test was used when expected cell counts were insufficient for the chi-square approximation.

Differences in inflammatory and adipokine biomarkers across chemerin tertiles were examined separately. Because five secondary biomarkers were simultaneously evaluated (i.e., tumor necrosis factor-α, retinol-binding protein 4, visfatin, vaspin, and omentin-1), *p*-values were adjusted using the Benjamini–Hochberg false-discovery-rate procedure.

The unadjusted association between circulating chemerin and PWV was initially evaluated using Spearman’s rank correlation coefficient. The independent association between chemerin and PWV was subsequently assessed using multivariable ordinary least-squares regression with heteroskedasticity-consistent HC3 standard errors. Chemerin was standardized, and the regression coefficient was expressed as the absolute difference in PWV, in m/s, associated with a one-standard-deviation increase in chemerin.

The multivariable model included age, sex, body mass index, mean arterial pressure, resting heart rate, current smoking status, and estimated glomerular filtration rate. Potential nonlinear associations of age and mean arterial pressure with PWV were modeled using centered natural cubic splines with four degrees of freedom. A possible nonlinear association between circulating chemerin and PWV was additionally explored by replacing the linear chemerin term with a centered natural cubic spline with four degrees of freedom; nonlinearity was assessed by a joint Wald test of the nonlinear spline components.

As a complementary analysis, adjusted PWV differences across tertiles of observed chemerin were estimated using the same covariate set. The lowest tertile was used as the reference category. A linear trend across tertiles was assessed by entering the chemerin tertile as an ordinal variable.

### 4.6. Chemerin–PWV Discordance Analysis

To investigate discordance between the circulating chemerin and vascular stiffness phenotypes, expected chemerin and expected PWV values were estimated separately.

Expected chemerin was modeled as a function of age, sex, body mass index, and estimated glomerular filtration rate. Age was modeled using a centered natural cubic spline with four degrees of freedom.

Expected PWV was modeled as a function of age, sex, body mass index, mean arterial pressure, resting heart rate, current smoking status, and estimated glomerular filtration rate. Age and mean arterial pressure were modeled using centered natural cubic splines with four degrees of freedom. The covariate sets were intentionally outcome-specific. The expected-chemerin model was designed to account for major demographic, adiposity-related, and renal determinants of circulating chemerin, whereas the mean arterial pressure, resting heart rate, and smoking status were additionally included in the expected-PWV model because of their direct relevance to vascular stiffness and hemodynamic loading. Thus, the primary residuals represented variation unexplained by the principal determinants selected for each phenotype. To assess the sensitivity of phenotype assignment to this asymmetric specification, the expected-chemerin model was additionally refitted using the same covariate set as the expected-PWV model, comprising age, sex, body mass index, mean arterial pressure, resting heart rate, smoking status, and estimated glomerular filtration rate, while otherwise preserving the original modeling strategy.

For each participant, chemerin and PWV residuals were calculated as the observed value minus the corresponding model-predicted value. A positive residual therefore indicated a value higher than expected given the included covariates, whereas a negative residual indicated a value lower than expected.

Chemerin and PWV residuals were independently divided into tertiles, generating a 3 × 3 discordance matrix. Tertiles were selected a priori as a compromise between identifying participants with relatively extreme residual values and retaining sufficient sample size for phenotype characterization. In addition, the middle tertile provided an intermediate zone, thereby avoiding the forced classification of participants with residual values close to the center of the distribution as either high or low, as would occur with a median-based split. Quartiles would provide a more restrictive definition of the extreme residual patterns but at the cost of substantially smaller groups. The middle tertiles were retained as an intermediate zone to reduce the classification of participants with residual values close to the distributional cut-off points as extreme phenotypes.

The two main residual patterns of interest were defined as follows: (i) higher-than-expected chemerin/lower-than-expected PWV, characterized by a chemerin residual in the highest tertile and a PWV residual in the lowest tertile, and (ii) higher-than-expected chemerin/higher-than-expected PWV, characterized by both residuals in the highest tertile. These definitions are purely descriptive of the relative position of each participant within the residual distributions and do not imply prognostic or cardiovascular-risk categories.

Clinical, metabolic, lifestyle, and inflammatory characteristics were compared between these two phenotypes using the Mann–Whitney U test for continuous variables and the chi-square or Fisher’s exact test for categorical variables. Standardized mean differences were also calculated to describe the magnitude of between-group differences independently of sample size.

Factors potentially associated with membership in the higher-than-expected chemerin/lower-than-expected PWV group were further examined using separate multivariable logistic regression models. The dependent variable was membership in this group rather than the higher-than-expected chemerin/higher-than-expected PWV group. Each candidate factor was entered individually and adjusted for age, sex, body mass index, mean arterial pressure, resting heart rate, current smoking status, and estimated glomerular filtration rate. Age and mean arterial pressure were modeled using natural cubic splines. Continuous candidate factors were standardized, and odds ratios were expressed per one-standard-deviation increase.

Because the phenotype-characterization analyses were exploratory and included multiple comparisons, both nominal *p*-values and Benjamini–Hochberg false-discovery-rate-adjusted q-values were reported. Nominal *p*-values refer to the unadjusted test-specific probabilities, whereas q-values account for multiple comparisons. Statistical tests were two-sided. Nominal associations were defined as *p* < 0.05, whereas exploratory findings were considered statistically robust only when the corresponding FDR-adjusted q-value was <0.05.

### 4.7. Sensitivity Analyses

The stability of the residual-based classification was assessed using 10-fold cross-fitting. The models for expected chemerin and expected PWV were fitted in nine folds and used to generate predicted values for participants in the held-out fold. This process was repeated until out-of-sample predicted values were available for all participants. Agreement between the original and cross-fitted chemerin tertiles, PWV tertiles, and 3 × 3 phenotype cells was then calculated. Cross-fitting was used exclusively to assess the internal statistical stability of phenotype assignment and was not intended as biological, prognostic, or clinical validation of the derived phenotypes.

To assess the sensitivity of the residual-based classification to the choice of cut-off points, the analysis was additionally repeated using two alternative schemes. First, chemerin and PWV residuals were dichotomized at their respective medians to generate a 2 × 2 classification. Second, residuals were divided into quartiles to generate a 4 × 4 classification, with the highest-chemerin/lowest-PWV and highest-chemerin/highest-PWV cells used as the corresponding extreme groups. The same clinical, metabolic, and inflammatory comparisons used for the primary phenotype characterization were repeated under both alternative classifications, including Benjamini–Hochberg false-discovery-rate correction.

To further assess the robustness of the primary chemerin–PWV association, the multivariable model was repeated with additional adjustment for known diabetes, known hypertension, lipid-lowering treatment, and TNF-α, considered individually. A separate model replaced body mass index with waist circumference as a measure of central adiposity. Finally, an extended sensitivity model simultaneously included known diabetes, known hypertension, lipid-lowering treatment, and TNF-α, with waist circumference replacing body mass index. All sensitivity models retained the remaining covariates and modeling specifications of the primary analysis.

## 5. Conclusions

In this community-based population, circulating chemerin was weakly associated with PWV in unadjusted analyses, but the relationship was attenuated and no longer statistically significant after adjustment for major clinical and hemodynamic determinants. Accordingly, there was no statistically significant independent association between circulating chemerin and central arterial stiffness in the overall population.

The residual-based analysis provided a secondary, hypothesis-generating perspective by identifying individual patterns of chemerin–PWV discordance that were not captured by the average regression coefficient. However, these data-derived patterns have not been externally or prospectively validated, and the clinical characteristics associated with them were exploratory and did not remain statistically significant after correction for multiple testing. They should therefore not be interpreted as clinically meaningful phenotypes at present. Future prospective studies should integrate isoform-specific chemerin measurements, sex-stratified analyses, assessment of local vascular and perivascular signaling, and longitudinal vascular outcomes to determine whether these residual patterns have biological or prognostic relevance.

## Figures and Tables

**Table 1:** Characteristics of the analytical sample according to tertiles of observed chemerin.

Variable	Overall (*n* = 1304)	Tertile 1 (*n* = 436)	Tertile 2 (*n* = 433)	Tertile 3 (*n* = 435)	*p*-Value	Available *n*
**Demographic and anthropometric characteristics**						
Age, years	58.0 [46.8–71.0]	57.0 [46.0–68.0]	58.0 [46.0–72.0]	59.0 [48.0–72.0]	0.239	1304
Women, *n* (%)	676 (51.8)	236 (54.1)	209 (48.3)	231 (53.1)	0.182	1304
Current smokers, *n* (%)	225 (17.3)	68 (15.6)	80 (18.5)	77 (17.7)	0.508	1304
Body mass index, kg/m^2^	26.3 [23.6–29.4]	26.0 [23.3–29.1]	26.3 [23.6–29.5]	26.4 [23.9–29.5]	0.302	1304
Waist circumference, cm	93.0 [83.0–101.0]	92.0 [83.0–101.0]	93.0 [84.0–102.0]	93.0 [83.5–101.0]	0.725	1297
**Hemodynamic and renal characteristics**						
Systolic blood pressure, mmHg	137 [126–152]	136 [125–153]	139 [128–152]	137 [126–151]	0.191	1304
Diastolic blood pressure, mmHg	72 [67–79]	72 [66–78]	73 [67–80]	72 [67–79]	0.177	1304
Mean arterial pressure, mmHg	94.3 [87.6–103.3]	92.7 [86.3–103.7]	95.3 [88.3–104.0]	94.7 [87.8–101.3]	0.171	1304
Heart rate, bpm	63 [56–70]	63 [56–69]	63 [57–70]	63 [56–70]	0.821	1304
eGFR, mL/min/1.73 m^2^	69.8 [60.4–80.6]	70.9 [62.5–81.2]	69.7 [59.6–81.2]	69.3 [60.1–79.1]	0.102	1304
**Metabolic characteristics**						
Triglycerides, mg/dL	102 [73–145]	98 [71–137]	107 [74–149]	102 [74–145]	0.086	1304
HDL cholesterol, mg/dL	50 [40–61]	50 [41–60]	51 [40–63]	50 [41–60]	0.315	1304
LDL cholesterol, mg/dL	139.4 [115.4–164.0]	142.0 [118.0–165.4]	135.5 [114.7–162.1]	140.6 [115.2–164.1]	0.180	1293
Fasting glucose, mg/dL	92 [85–101]	91 [85–99]	92 [85–102]	92 [85–102]	0.252	1304
**Clinical history**						
Known hypertension, *n* (%)	436 (34.1)	139 (32.3)	137 (32.6)	160 (37.2)	0.239	1280
Known diabetes, *n* (%)	100 (7.8)	31 (7.2)	34 (8.1)	35 (8.2)	0.845	1276
**Treatments**						
Antihypertensive treatment, *n* (%)	444 (34.0)	138 (31.7)	147 (33.9)	159 (36.6)	0.312	1304
Lipid-lowering treatment, *n* (%)	255 (20.2)	76 (18.0)	94 (22.3)	85 (20.3)	0.287	1262
Glucose-lowering treatment, *n* (%)	56 (4.5)	16 (3.8)	18 (4.3)	22 (5.4)	0.548	1244
**Vascular and biomarker measurements**						
Pulse wave velocity, m/s	8.7 [7.6–10.2]	8.5 [7.5–9.9]	8.8 [7.7–10.4]	8.9 [7.7–10.3]	0.019	1304
Chemerin, ng/mL	326.93 [250.59–393.43]	224.28 [189.43–250.65]	326.93 [297.20–350.28]	422.36 [393.47–455.81]	<0.001	1304
TNF-α, pg/mL	6.28 [3.68–9.19]	5.99 [3.25–9.11]	5.98 [3.80–8.70]	7.13 [4.30–9.79]	<0.001	1304

Data are presented as median [interquartile range] or number (percentage). *p*-values were calculated using the Kruskal–Wallis test for continuous variables and the chi-square test for categorical variables. The cut-off points between chemerin tertiles were 272.65 ng/mL and 371.77 ng/mL. TNF-α is included as the principal secondary inflammatory biomarker. The available sample size is reported because variables not included in the complete-case selection could contain missing values. Abbreviations: eGFR, estimated glomerular filtration rate; HDL, high-density lipoprotein; LDL, low-density lipoprotein; PWV, pulse wave velocity; TNF-α, tumor necrosis factor-α. Median chemerin concentrations were 224.28 ng/mL [189.43–250.65] in the lowest tertile, 326.93 ng/mL [297.20–350.28] in the middle tertile, and 422.36 ng/mL [393.47–455.81] in the highest tertile.

**Table 2:** Clinical, metabolic, and inflammatory characteristics of the contrasting high-chemerin residual groups.

Variable	Higher-than-Expected Chemerin/Lower-than-Expected PWV (*n* = 132)	Higher-than-Expected Chemerin/Higher-than-Expected PWV (*n* = 151)	SMD	Nominal *p*-Value	FDR-Adjusted q-Value
Age, years	64.0 [51.0–74.0]	59.0 [47.0–71.0]	0.225	0.052	0.465
Women, *n* (%)	75 (56.8)	76 (50.3)	0.130	0.275	0.682
Body mass index, kg/m^2^	26.85 [23.43–29.74]	25.89 [23.76–29.07]	0.052	0.366	0.763
Waist circumference, cm	94.50 [86.00–101.25]	91.00 [83.25–100.00]	0.170	0.081	0.465
Current smokers, *n* (%)	15 (11.4)	28 (18.5)	−0.202	0.093	0.465
Mean arterial pressure, mmHg	95.35 [88.22–105.40]	94.70 [87.35–100.50]	0.195	0.196	0.622
Heart rate, bpm	63.0 [56.0–71.0]	64.0 [57.0–71.5]	−0.091	0.684	0.855
eGFR, mL/min/1.73 m^2^	69.25 [57.35–79.48]	70.15 [60.34–79.19]	−0.168	0.199	0.622
Fasting glucose, mg/dL	93.0 [87.0–103.0]	92.0 [84.0–103.0]	0.012	0.485	0.783
Triglycerides, mg/dL	102.5 [80.0–141.5]	96.0 [69.0–144.0]	0.040	0.491	0.783
HDL cholesterol, mg/dL	52.0 [41.0–61.5]	50.0 [42.0–60.0]	0.065	0.629	0.855
LDL cholesterol, mg/dL	141.4 [116.1–166.9]	135.8 [111.8–160.0]	0.263	0.084	0.465
LAP index	37.53 [22.59–60.63]	35.09 [18.70–52.82]	0.164	0.119	0.496
Hepatic steatosis index	37.77 [33.50–41.99]	36.72 [33.70–41.48]	0.025	0.748	0.857
Known hypertension, *n* (%)	51 (39.2)	53 (35.3)	0.081	0.501	0.783
Known diabetes, *n* (%)	7 (5.4)	18 (12.2)	−0.244	0.047	0.465
Antihypertensive treatment, *n* (%)	52 (39.4)	53 (35.1)	0.089	0.456	0.783
Lipid-lowering treatment, *n* (%)	25 (19.7)	37 (25.3)	−0.136	0.266	0.682
Uric acid, mg/dL	5.25 [4.38–6.00]	5.20 [4.40–6.00]	−0.094	0.788	0.857
Fasting plasma insulin, µU/mL	15.54 [13.56–18.68]	15.40 [13.46–19.84]	−0.052	0.914	0.949
TNF-α, pg/mL	7.04 [3.91–9.47]	6.59 [3.96–9.38]	0.041	0.777	0.857
RBP4, µg/mL	45.80 [36.40–63.42]	46.89 [36.77–62.22]	0.008	0.949	0.949
Visfatin, ng/mL	5.29 [4.52–6.81]	5.56 [4.47–7.08]	−0.036	0.649	0.855
Vaspin, ng/mL	0.55 [0.42–0.95]	0.55 [0.44–1.20]	−0.146	0.300	0.682
Omentin-1, ng/mL	11.68 [8.84–15.80]	12.80 [9.20–15.65]	−0.033	0.666	0.855

The higher-than-expected chemerin/lower-than-expected PWV group was defined by a chemerin residual in the highest tertile and a PWV residual in the lowest tertile; the higher-than-expected chemerin/higher-than-expected PWV group was defined by both residuals in the highest tertile. Data are presented as median [interquartile range] or number (percentage). Nominal *p*-values are unadjusted for multiple comparisons and were obtained using the Mann–Whitney U test for continuous variables and the chi-square or Fisher’s exact test for categorical variables, as appropriate. FDR-adjusted q-values were calculated using the Benjamini–Hochberg procedure across the comparisons shown. A positive standardized mean difference indicates a higher value or prevalence in the higher-than-expected chemerin/lower-than-expected PWV group. Abbreviations: eGFR, estimated glomerular filtration rate; FDR, false discovery rate; HDL, high-density lipoprotein; LAP, lipid accumulation product; LDL, low-density lipoprotein; PWV, pulse wave velocity; RBP4, retinol-binding protein 4; SMD, standardized mean difference; TNF-α, tumor necrosis factor-α.

**Table 3:** Factors associated with higher-than-expected chemerin/lower-than-expected PWV versus higher-than-expected chemerin/higher-than-expected PWV classification (*n* = 283).

Candidate Factor	Adjusted OR	95% CI	Nominal *p*-Value	FDR-Adjusted q-Value	Scale
Waist circumference	1.77	1.10–2.84	0.018	0.225	Per 1-SD increase
Known diabetes	0.32	0.12–0.87	0.025	0.225	Yes vs. no
LDL cholesterol	1.28	1.00–1.64	0.050	0.277	Per 1-SD increase
Lipid-lowering treatment	0.54	0.28–1.06	0.073	0.277	Yes vs. no
Vaspin	0.80	0.63–1.02	0.077	0.277	Per 1-SD increase
LAP index	1.28	0.93–1.76	0.126	0.378	Per 1-SD increase
Current smoking	0.63	0.31–1.28	0.202	0.519	Yes vs. no
Visfatin	0.91	0.71–1.16	0.437	0.830	Per 1-SD increase
Uric acid	0.88	0.64–1.22	0.459	0.830	Per 1-SD increase
TNF-α	1.09	0.85–1.40	0.497	0.830	Per 1-SD increase
Fasting plasma insulin	0.92	0.72–1.17	0.507	0.830	Per 1-SD increase
Hepatic steatosis index	1.16	0.68–2.01	0.584	0.867	Per 1-SD increase
Antihypertensive treatment	0.86	0.46–1.59	0.626	0.867	Yes vs. no
HDL cholesterol	0.94	0.72–1.25	0.687	0.883	Per 1-SD increase
Triglycerides	1.04	0.81–1.35	0.738	0.886	Per 1-SD increase
Fasting glucose	0.97	0.74–1.26	0.795	0.894	Per 1-SD increase
Omentin-1	0.98	0.76–1.26	0.883	0.907	Per 1-SD increase
RBP4	0.99	0.77–1.26	0.907	0.907	Per 1-SD increase

Odds ratios represent the odds of belonging to the higher-than-expected chemerin/lower-than-expected PWV group rather than the higher-than-expected chemerin/higher-than-expected PWV group. Each candidate factor was evaluated in a separate logistic regression model adjusted for age, sex, body mass index, mean arterial pressure, resting heart rate, current smoking status, and estimated glomerular filtration rate. Age and mean arterial pressure were modeled using centered natural cubic splines. Continuous candidate factors were standardized before analysis. Nominal *p*-values are unadjusted for multiple comparisons. FDR-adjusted q-values were calculated using the Benjamini–Hochberg procedure across the candidate factors shown. Abbreviations: CI, confidence interval; FDR, false discovery rate; HDL, high-density lipoprotein; LAP, lipid accumulation product; LDL, low-density lipoprotein; OR, odds ratio; RBP4, retinol-binding protein 4; SD, standard deviation; TNF-α, tumor necrosis factor-α.

## Data Availability

Due to administrative regulations within the local health system and specific restrictions outlined in the protocol approved by the institutional Ethics Review Board, the data supporting the findings of this study are not publicly available but are obtainable from the corresponding author upon reasonable request.

## Supplementary Materials

The following supporting information can be downloaded at https://www.mdpi.com/article/10.3390/ijms27188264/s1.

## Abbreviations

The following abbreviations are used in this manuscript:

BHS	Brisighella Heart Study
BMI	Body mass index
CCRL2	C-C motif chemokine receptor-like 2
CI	Confidence interval
CKD-EPI	Chronic Kidney Disease Epidemiology Collaboration
CMKLR1	Chemokine-like receptor 1
CV	Coefficient of variation
eGFR	Estimated glomerular filtration rate
ELISA	Enzyme-linked immunosorbent assay
FDR	False discovery rate
GPR1	G protein-coupled receptor 1
HC3	Heteroskedasticity-consistent type 3 standard errors
HDL	High-density lipoprotein
LAP	Lipid accumulation product
LDL	Low-density lipoprotein
OR	Odds ratio
PVAT	Perivascular adipose tissue
PWV	Pulse wave velocity
RARRES2	Retinoic acid receptor responder 2
RBP4	Retinol-binding protein 4
SD	Standard deviation
SMD	Standardized mean difference
TNF-α	Tumor necrosis factor alpha

## References

[1] Onofrei V.A., Zamfir C.L., Anisie E., Ceasovschih A., Constantin M., Mitu F., Adam C.A., Grigorescu E.D., Petroaie A.D., Timofte D. (2023). Determinants of arterial stiffness in patients with morbid obesity: The role of echocardiography and carotid ultrasound imaging. Medicina.

[2] Li H., Zhan J., Liao B., Wang Y., Liu Y. (2019). Plasma levels of adiponectin and chemerin are associated with early stage of atherosclerosis in older adults with type 2 diabetes mellitus. Aging Med..

[3] Onofrei V.A., Anisie E., Zamfir C.L., Ceasovschih A., Constantin M., Mitu F., Grigorescu E.D., Petroaie A.D., Timofte D.V. (2023). Role of chemerin and perivascular adipose tissue characteristics on cardiovascular risk assessment by arterial stiffness markers in patients with morbid obesity. J. Clin. Med..

[4] Macvanin M.T., Rizzo M., Radovanovic J., Sonmez A., Paneni F., Isenovic E.R. (2022). Role of chemerin in cardiovascular diseases. Biomedicines.

[5] Ahmed A., Bibi A., Valoti M., Fusi F. (2023). Perivascular adipose tissue and vascular smooth muscle tone: Friends or foes?. Cells.

[6] Man A.W.C., Xia N., Li H. (2025). Vascular effects of perivascular adipose tissue-derived chemerin in obesity-associated cardiovascular disease. Cardiovasc. Diabetol..

[7] Tan L., Lu X., Danser A.H.J., Verdonk K. (2023). The role of chemerin in metabolic and cardiovascular disease: A literature review of its physiology and pathology from a nutritional perspective. Nutrients.

[8] Wabel E., Orr A., Flood E.D., Thompson J.M., Xie H., Demireva E.Y., Abolibdeh B., Honke Hulbert D., Mullick A.E., Garver H. (2023). Chemerin is resident to vascular tunicas and contributes to vascular tone. Am. J. Physiol. Heart Circ. Physiol..

[9] Wabel E., Garver H., Krieger-Burke T., Xie F., Zeng L., Mullick A., Fink G.D., Watts S.W. (2026). Chemerin knockout reveals sex difference in the role of chemerin in blood pressure and vascular remodeling. Am. J. Physiol. Heart Circ. Physiol..

[10] Aydın K., Canpolat U., Akın Ş., Dural M., Karakaya J., Aytemir K., Özer N., Gürlek A. (2016). Chemerin is not associated with subclinical atherosclerosis markers in prediabetes and diabetes. Anatol. J. Cardiol..

[11] Parlee S.D., Ernst M.C., Muruganandan S., Sinal C.J., Goralski K.B. (2010). Serum chemerin levels vary with time of day and are modified by obesity and tumor necrosis factor-α. Endocrinology.

[12] Gu P., Cheng M., Hui X., Lu B., Jiang W., Shi Z. (2015). Elevating circulation chemerin level is associated with endothelial dysfunction and early atherosclerotic changes in essential hypertensive patients. J. Hypertens..

[13] Parlee S.D., McNeil J.O., Muruganandan S., Sinal C.J., Goralski K.B. (2012). Elastase and tryptase govern TNFα-mediated production of active chemerin by adipocytes. PLoS ONE.

[14] Schinzari F., Montenero R., Cardillo C., Tesauro M. (2025). Chemerin is the adipokine linked with endothelin-dependent vasoconstriction in human obesity. Biomedicines.

[15] Neves K.B., Nguyen Dinh Cat A., Lopes R.A., Rios F.J., Anagnostopoulou A., Lobato N.S., de Oliveira A.M., Tostes R.C., Montezano A.C., Touyz R.M. (2015). Chemerin Regulates Crosstalk Between Adipocytes and Vascular Cells Through Nox. Hypertension.

[16] Watts S.W., Dorrance A.M., Penfold M.E., Rourke J.L., Sinal C.J., Seitz B., Sullivan T.J., Charvat T.T., Thompson J.M., Burnett R. (2013). Chemerin connects fat to arterial contraction. Arterioscler. Thromb. Vasc. Biol..

[17] Ahmed N.A., Hussein A.A., Khalil R.G., Yassin N.Y.S., Abdelaziz M.A., Geddawy A.I., Ahmed O.M. (2026). Chemerin normal function and roles in metabolic and nonmetabolic disorders: An up-to-date comprehensive review. Biochem. Res. Int..

[18] Sell H., Laurencikiene J., Taube A., Eckardt K., Cramer A., Horrighs A., Arner P., Eckel J. (2009). Chemerin is a novel adipocyte-derived factor inducing insulin resistance in primary human skeletal muscle cells. Diabetes.

[19] Gasbarrino K., Hafiane A., Gianopoulos I., Zheng H., Mantzoros C.S., Daskalopoulou S.S. (2023). Relationship between circulating adipokines and cholesterol efflux in subjects with severe carotid atherosclerosis. Metabolism.

[20] Cicero A.F.G., Fogacci F., Derosa G., D’Angelo A., Ventura F., Rizzoli E., D’Addato S., Borghi C. (2022). on behalf of the Brisighella Heart Study Group. Lipoprotein(a) serum levels predict pulse wave velocity in subjects in primary prevention for cardiovascular disease with large Apo(a) isoforms: Data from the Brisighella Heart Study. Biomedicines.

[21] Cicero A.F.G., Gitto S., Fogacci F., Rosticci M., Giovannini M., D’Addato S., Andreone P., Borghi C., Brisighella Heart Study Group (2018). Fatty liver index is associated to pulse wave velocity in healthy subjects: Data from the Brisighella Heart Study. Eur. J. Intern. Med..

[22] Cicero A.F.G., Fogacci F., D’Addato S., Grandi E., Rizzoli E., Borghi C., on behalf of the Brisighella Heart Study (2023). Self-reported coffee consumption and central and peripheral blood pressure in the cohort of the Brisighella Heart Study. Nutrients.

[23] Mancia G., Kreutz R., Brunström M., Burnier M., Grassi G., Januszewicz A., Muiesan M.L., Tsioufis K., Agabiti-Rosei E., Algharably E.A.E. (2023). 2023 ESH Guidelines for the management of arterial hypertension: The Task Force for the management of arterial hypertension of the European Society of Hypertension. Endorsed by the International Society of Hypertension and the European Renal Association. J. Hypertens..

[24] Cicero A.F.G., Fogacci F., Giovannini M., Grandi E., D’Addato S., Borghi C. (2019). Brisighella Heart Study Group. Interaction between low-density lipoprotein-cholesterolaemia, serum uric level and incident hypertension: Data from the Brisighella Heart Study. J. Hypertens..

[25] Levey A.S., Stevens L.A., Schmid C.H., Zhang Y.L., Castro A.F., Feldman H.I., Kusek J.W., Eggers P., Van Lente F., Greene T. (2009). A new equation to estimate glomerular filtration rate. Ann. Intern. Med..

[26] Hida K., Wada J., Eguchi J., Zhang H., Baba M., Seida A., Hashimoto I., Okada T., Yasuhara A., Nakatsuka A. (2005). Visceral adipose tissue-derived serine protease inhibitor: A unique insulin-sensitizing adipocytokine in obesity. Proc. Natl. Acad. Sci. USA.

[27] Körner A., Garten A., Blüher M., Tauscher R., Kratzsch J., Kiess W. (2007). Molecular characteristics of serum visfatin and differential detection by immunoassays. J. Clin. Endocrinol. Metab..

[28] de Souza Batista C.M., Yang R.Z., Lee M.J., Glynn N.M., Yu D.Z., Pray J., Ndubuizu K., Patil S., Schwartz A., Kligman M. (2007). Omentin plasma levels and gene expression are decreased in obesity. Diabetes.

[29] Hickson S.S., Butlin M., Broad J., Avolio A.P., Wilkinson I.B., McEniery C.M. (2009). Validity and repeatability of the Vicorder apparatus: A comparison with the SphygmoCor device. Hypertens. Res..

[30] Milan A., Zocaro G., Leone D., Tosello F., Buraioli I., Schiavone D., Veglio F. (2019). Current assessment of pulse wave velocity: Comprehensive review of validation studies. J. Hypertens..

[31] McGreevy C., Barry M., Bennett K., Williams D. (2013). Repeatability of the measurement of aortic pulse wave velocity in the clinical assessment of arterial stiffness in community-dwelling older patients using the Vicorder^®^ device. Scand. J. Clin. Lab. Investig..

