# Supplementary material for: Chemerin and Arterial Stiffness in the Brisighella Heart Study: A Residual-Based Discordance Analysis
Source: Int J Mol Sci. 2026 Sep 16;27(18):8264. doi: 10.3390/ijms27188264 (PMC13607014; doi:10.3390/ijms27188264)
Supplement: Supplementary file 1 [file ijms-27-08264-s001.zip › ijms-4497303-supplementary.pdf]

Supplementary Table S1. Comparison of main characteristics between participants included in and excluded from the analytical sample.

Variable	Included participants	Excluded participants	Absolute SMD
Age, years	58.0 [46.8–71.0]	59.0 [48.0–72.0]	0.071
Women, n (%)	676 (51.8)	189 (57.3)	0.109
Body mass index, kg/m²	26.3 [23.6–29.4]	25.8 [23.3–29.5]	0.061
Current smokers, n (%)	225 (17.3)	60 (18.8)	0.039
Mean arterial pressure, mmHg	94.3 [87.6–103.3]	98.2 [90.6–108.1]	0.284
Heart rate, bpm	63 [56–70]	64 [55–71]	0.037
eGFR, mL/min/1.73 m²	69.8 [60.4–80.6]	71.4 [60.5–80.6]	0.003
Known diabetes, n (%)	100 (7.8)	26 (8.1)	0.010

Data are presented as median [interquartile range] or number (percentage), as appropriate. Because missing data contributed to participant exclusion, the number of available observations varied across characteristics in the excluded group. Absolute standardized mean differences (SMDs) were used to quantify between-group differences. Abbreviations: BMI, body mass index; eGFR, estimated glomerular filtration rate; MAP, mean arterial pressure; SMD, standardized mean difference.

Supplementary Table S2. Inflammatory and adipokine profile according to tertiles of observed chemerin.

Biomarker	Overall (n = 1,304)	Tertile 1 (n = 436)	Tertile 2 (n = 433)	Tertile 3 (n = 435)	Nominal p value	FDR-adjusted q value
TNF-α, pg/mL	6.28 [3.68–9.19]	5.99 [3.25–9.11]	5.98 [3.80–8.70]	7.13 [4.30–9.79]	<0.001	0.001
RBP4, μg/mL	48.31 [36.57–66.93]	54.00 [39.50–69.43]	47.25 [35.52–65.90]	45.60 [35.55–65.26]	<0.001	<0.001

Visfatin, ng/mL	5.70 [4.57–7.38]	5.95 [4.83–7.52]	5.92 [4.57–7.70]	5.42 [4.44–6.88]	<0.001	<0.001
Vaspin, ng/mL	0.56 [0.42–1.04]	0.56 [0.42–1.12]	0.56 [0.40–1.00]	0.56 [0.42–0.98]	0.534	0.534
Omentin-1, ng/mL	12.77 [8.84–16.48]	12.65 [8.62–16.06]	12.77 [8.85–16.85]	12.78 [8.88–16.27]	0.304	0.379

Data are presented as median [interquartile range]. P values were calculated using the Kruskal–Wallis test. q values were obtained using the Benjamini–Hochberg false-discovery-rate procedure across the five biomarkers shown. Abbreviations: FDR, false discovery rate; RBP4, retinol-binding protein 4; TNF-α, tumor necrosis factor-α.

Supplementary Table S3. Sensitivity analyses of the chemerin–PWV association and residual-based phenotype classification

**Sensitivity analysis**	**Analysis n**	**H/L group, n**	**H/H group, n**	**Agreement with primary classification, %**	**H/L retained, %**	**H/H retained, %**	**β, m/s**	**p value**	**Minimum q-FDR**
10-fold cross-fitting	1,304	—	—	Chemerin tertile: 98.3; PWV tertile: 97.2; 3 × 3 cell: 95.6	—	—	—	—	—
PWV <3 m/s retained	1,306	—	—	—	—	—	0.092	0.136	—
Harmonized covariate set	1,304	133	151	Chemerin tertile: 94.8; 3 × 3 cell: 94.8	97.7	96.0	—	—	—
Median-based 2 × 2 classification	1,304	313	339	—	—	—	—	—	0.672
Quartile-based 4 × 4 classification	1,304	74	81	—	—	—	—	—	0.670

Data are presented as sample size, group size, percentage agreement, regression coefficient, p value, or minimum false-discovery-rate-adjusted q value, as appropriate. H/L denotes the group with a chemerin residual in the highest category and a PWV residual in the lowest category; H/H denotes the group with both chemerin and PWV residuals in the highest category. In the primary analysis, residuals were divided into tertiles to generate a 3 × 3 classification. The 10-fold cross-fitting analysis assessed agreement between the original and out-of-sample residual classifications. In the harmonized-covariate analysis, the expected-chemerin model was refitted using the same covariates as the expected-PWV model: age, sex, body mass index, mean arterial pressure, resting heart rate, smoking status, and estimated glomerular filtration rate. For the threshold-sensitivity analyses, the residuals from the primary prediction models were retained unchanged and

only the classification cut-points were modified. Median-based classification generated a 2 × 2 matrix, whereas quartile-based classification generated a 4 × 4 matrix. Clinical, metabolic, and inflammatory comparisons between the H/L and H/H groups were repeated using the same procedures as in the primary analysis, including Benjamini–Hochberg false-discovery-rate correction. The analysis retaining PWV values <3 m/s used the same covariates and model specifications as the primary continuous chemerin–PWV analysis. Abbreviations: FDR, false discovery rate; PWV, pulse wave velocity.

Supplementary Table S4. Sensitivity analyses of the association between circulating chemerin and pulse wave velocity after additional covariate adjustment.

Analysis	n	β per 1-SD chemerin, m/s	95% CI	p value
Primary model	1,304	0.093	−0.026 to 0.212	0.126
Additional adjustment for known diabetes	1,276	0.082	−0.037 to 0.201	0.176
Additional adjustment for known hypertension	1,280	0.090	−0.031 to 0.211	0.144
Waist circumference replacing BMI	1,297	0.086	−0.034 to 0.206	0.162
Additional adjustment for lipid-lowering treatment	1,262	0.102	−0.020 to 0.223	0.100
Additional adjustment for TNF-α	1,304	0.096	−0.021 to 0.212	0.109
Extended sensitivity model*	1,214	0.091	−0.031 to 0.213	0.142

β coefficients represent the difference in PWV associated with a one-standard-deviation increase in circulating chemerin. The primary model was adjusted for age, sex, body mass index, mean arterial pressure, resting heart rate, smoking status, and estimated glomerular filtration rate. Age and mean arterial pressure were modeled using centered natural cubic splines with four degrees of freedom. Sensitivity analyses additionally adjusted for the factor specified in each row. In the waist-circumference analysis, waist circumference replaced body mass index. *The extended sensitivity model included age, sex, waist circumference, mean arterial pressure, resting heart rate, smoking status, estimated glomerular filtration rate, known diabetes, known hypertension, lipid-lowering treatment, and TNF-α. Heteroskedasticity-consistent HC3 standard errors were used throughout. Sample size varied because no missing-data imputation was performed. Abbreviations: BMI, body mass index; CI, confidence interval; eGFR, estimated glomerular filtration rate; PWV, pulse wave velocity; SD, standard deviation; TNF-α, tumor necrosis factor-α.
